# A Study of the Periods of Incubation of the Communicable Diseases

**Published:** 1882-08

**Authors:** Benjamin Ward Richardson


### Selections.

A STUDY OF THE PERIODS OF INCUBATION OF THE COMMUNICABLE DISEASES. BY BENJAMIN WARD RICHARDSON, M.D., F.R.C.P., F.R.S. (Continued from page 107, July number.)

The diseases of the first group are fairly steady in respect to incubation, but it may by some be raised as a question whether catarrh, either superficial or cellular, should be classed amongst the communicable diseases at all. On this point I am very interested to hear the opinion of this Society. That catarrh may be excited without communication I know, and it may be that it is always due to a general influence.

Of the diseases of the second or third group, exceptions may be adduced in which the disease may have a shorter incubation, in particular cases, than is assigned in the classification. Such exceptions must be admitted, and my own case of scarlet fever, quoted by Murchison, shows for this disease, occasionally a very brief incubation,—six hours at longest.

Others may question whether rosalia idiopathica has a period of incubation at all, and whether it is not more closely allied to those affections attended with rash, which, like urticaria, are induced by improper food, than to scarlet fever. I attended once, with Murchison, a case that terminated fatally from occurence of bullæ in the glottis,—coming on during convalesence,—in which we never determined whether the affection, which commenced like an attack of acute urticaria, was roseolous or not. The eruption became like that of scarlet fever, with large white blotches over the surface, and in all respects yielded the form of disease which Mason Good designated rosalia parithmitica.

I would prefer, for my part, to classify these as doubtful cases, and at the same time leave them open for further study.

The diseases of the third and fourth groups will be accepted as fairly steady. Relapsing fever, as I knew it in 1847, had a longer period than five days of incubation, but by inoculation it is said to have five days.

In the last group syphilis will be accepted as having the long incubation of forty days. But hydrophobia is the most difficult disease to define. It may have a few days of incubation only. It may have months or even years.

These considerations lead up to the third question with which I opened this paper in respect to differences of opinion on periods of incubation. Do these differences depend on fault of observation, or on the circumstance that there are, in the same diseases, varying periods of incubation, the variations being under no defined rule?

My answer to this question is, that the differences of opinion so often professionally expressed, and which strike the general public with such wonder, are due rather to want of uniformity of observation than to variation of natural phenomena.

This I trace to four sets of causes—

1. That we have never yet agreed altogether as to the first sign that indicates the close of incubation.

2. That until lately we have not ventured to classify, so that there has been no clear grouping of diseases by the test of incubation.

3. That we have been accustomed to consider exceptional phenomena of incubation as more common and important than they really are.

4. That we have been too much accustomed to connect diseases which are most disimilar in actual fact too closely because of certain similarities which do not really affect the question when all the facts are known.

Now that we are coming nearer to the truth we may hope to classify the exceptions, the nature of which is included under the next heading of my paper. Upon what circumstances do the exceptional periods of incubation of the communicable diseases occur, and why do they occur?

We may assume that a difference in physical property is the reason of the difference of action of the distinct poison of the defined communicable diseases. But when we are dealing with a single poison,—with the poison of scarlet fever, for instance,—the difficulty commences. We can scarcely conceive that the poison changes in character and operation; and we are, therefore, obliged to trace out its effects through the means by which it comes into operation.

In studying this difficulty we light at once upon one useful fact. We know, of a certainty, that a poison which will affect, both by inoculation and by absorption through the lungs or alimentary surface, acts according to the circumstance of introduction in distinctly different periods. In the case of small pox, it acts one-fifth more quickly when it is inoculated than when it is absorbed through membrane. There would, therefore, probably in every case of absorption, be a difference in favor of rapidity of action, if the poison should be distributed over an injured or abraded surface, so as to inoculate such a surface in transit.

It is fair again to assume that the poison may be inhaled in a form of diffusion more ready for absorption in some cases than in others. But the great cause of difference is, I suspect, in individual susceptibility to the action of the poison, and it may be to the different influence of the same poison under different times and conditions of life. With this part of our study we have to unravel all the questions connected with the course of the communicable diseases in relation to seasons. That we should be more susceptible to the action of the poisons of the spreading diseases at those seasons of the year when there is excessive waste of bodily structure, and that we should be less susceptible to them in genial seasons when there is a balance in favor of nutrition is not a strange physiological pathological phenomenon. That we are more susceptible at the one period and less so at the other is, I suspect, more than a mere coincidence.

Here, however, I come to a point in theory which must be reserved for experimental development and exposition.

I turn now lastly, and briefly, to the practical sides of the subject in hand, and these are many. I will allude to three.

If we could arrive at definite evidence on which we could all rest, that the incubation of a disease is produced by the occurrence of a certain indication, we could account for much that seems now to be mysterious. For example, in vaccinia can it be that, trusting entirely to the local phenomena, we sometimes have an apparently good vaccination in which the disease never has incubated at all? If that be so, there is a reason why the person who seems to have been thoroughly vaccinated, should still remain susceptible to small-pox. The suggestion, though it stands alone, is all-sufficient to indicate the practical importance of delivering incubation before pronouncing as to the protection afforded by some, but not all, the symptoms of a communicable affection.

The division of diseases of the communicable type into different classes or groups, according to the stage of incubation presented is, I think, a practical acquirement of the most important every-day value, both in curative and preventive science. Dr. Squire has not in the least degree over estimated this part of modern knowledge. It is quite true, as he shows, that to know when to retain at home a sick person laboring under communicable disease, when to send him from home, and when to separate him from the actual or presumably unaffected, is a direction of practical skill as eminently creditable to the profession of medicine as it is useful to the public. To remove the healthy from the affected is good practice in the case of all the diseases of the first and second of the groups I have given, *i.e.*, of the quick and short incubation series. To remove in the third series may be sound practice. To remove in the fourth, or long incubation class, is less important, and may be a practice that is quite at fault and prejudicial.

Lastly, from the study of the incubation period we learn how to assign the best means of disposing of the affected during convalescence. It seems to be a true reading of natural phenomena that the diseases of short incubation have a prolonged convalescence, and remain long as sources of communication; while the diseases which show a long incubation give a quicker convalescence, and a more rapid freedom from danger as sources of communication. Here, then, is a practical lesson that cannot well be over-estimated. Keep your cases of short incubation at home: they will infect others and be themselves injured by removal.—*Medical Press and Circular.*

